# Supplementary material for: Effects of Trichoderma harzianum combined with Phanerochaete chrysosporium on lignin degradation and humification during chicken manure and rice husk composting
Source: Front Microbiol. 2025 Feb 28;16:1515931. doi: 10.3389/fmicb.2025.1515931 (PMC11906335; doi:10.3389/fmicb.2025.1515931)
Supplement: Supplementary file 1 [file Data_Sheet_1.zip › 6.Function/All/FunGuild/l2/heatmap_sample.pdf]

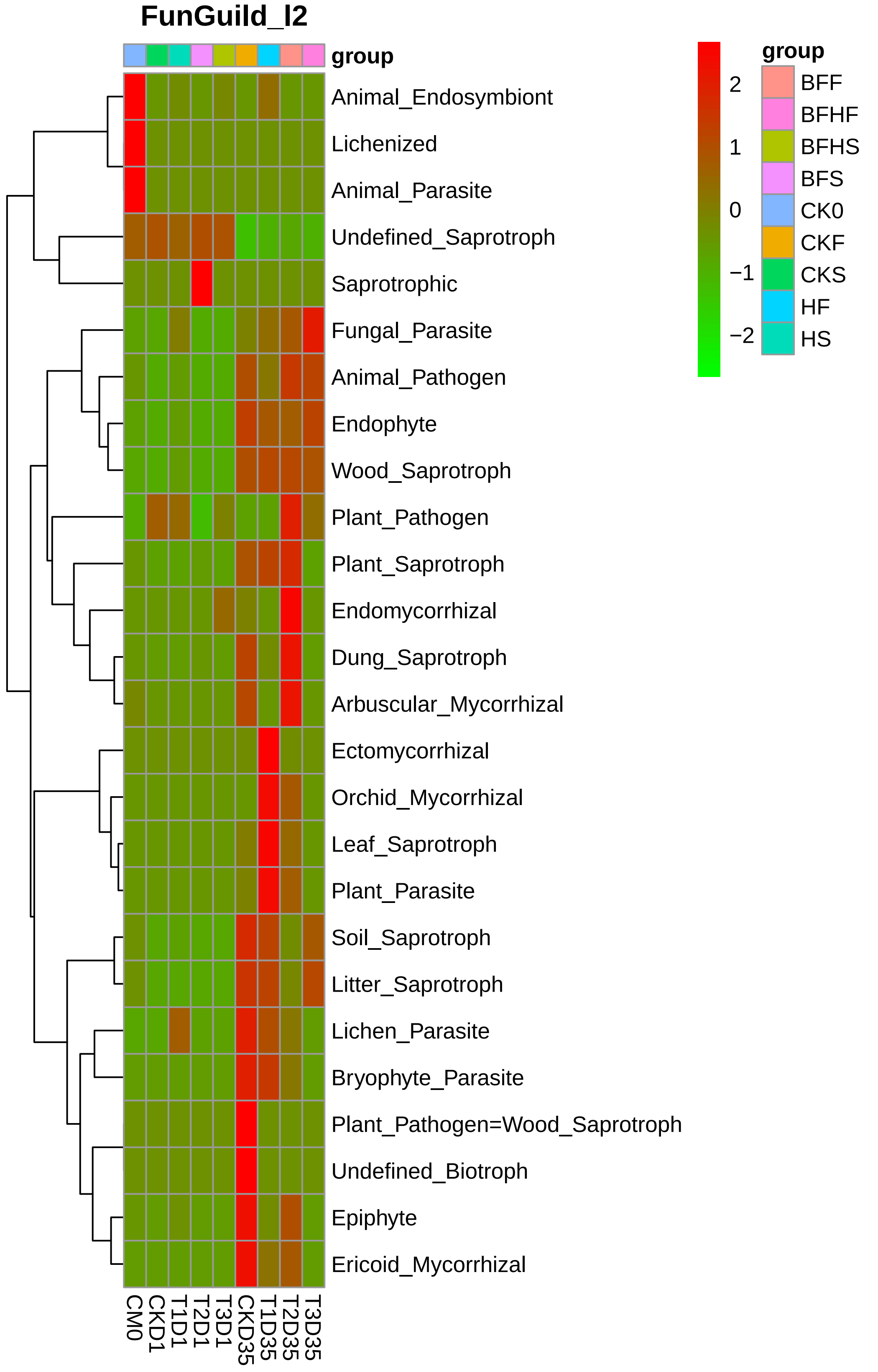
FunGuild_l2
group
Animal_Endosymbiont
Lichenized
Animal_Parasite
Undefined_Saprotroph
Saprotrophic
Fungal_Parasite
Animal_Pathogen
Endophyte
Wood_Saprotroph
Plant_Pathogen
Plant_Saprotroph
Endomycorrhizal
Dung_Saprotroph
Arbuscular_Mycorrhizal
Ectomycorrhizal
Orchid_Mycorrhizal
Leaf_Saprotroph
Plant_Parasite
Soil_Saprotroph
Litter_Saprotroph
Lichen_Parasite
Bryophyte_Parasite
Plant_Pathogen=Wood_Saprotroph
Undefined_Biotroph
Epiphyte
Ericoid_Mycorrhizal
CM0
CKD1
T1D1
T2D1
T3D1
CKD35
T1D35
T2D35
T3D35
2
1
0
−1
−2
group
BFF
BFHF
BFHS
BFS
CK0
CKF
CKS
HF
HS
